# Growth effect on liver fatty acid composition of damselfishes genus *Abudefduf* collected in coral reef habitats of the Malaysian South China Sea

**DOI:** 10.1186/s40064-015-0862-5

**Published:** 2015-02-10

**Authors:** Takaomi Arai, Razikin Amalina, Zainudin Bachok

**Affiliations:** Institute of Oceanography and Environment, Universiti Malaysia Terengganu, 21030 Kuala Terengganu, Terengganu Malaysia; School of Marine Science and Environment, Universiti Malaysia Terengganu, 21030 Kuala Terengganu, Terengganu Malaysia

**Keywords:** Coral fish, Diet, Habitat, *Abudefduf*, Migration, South China Sea

## Abstract

In order to understand feeding ecology, habitat use and migration of coral reef fish, fatty acid composition was examined in damselfish species *Abudefduf bengalensis* and *A. sexfasciatus* collected in the Malaysian South China Sea. Proportions of saturated fatty acids (SAFA) ranged from 49.5% to 74.2%, with the highest proportions in fatty acids, the second highest was monounsaturated fatty acids (MUFA) ranged from 21.4% to 47.4% and the proportion of polyunsaturated fatty acids (PUFA) was the lowest ranged from 3.1% to 6.0%. Palmitic acid (16:0) was the most common in SAFA, oleic acid (C18:1ω9c) was the dominant in MUFA and linolenic acid (C18:3n3) showed the highest proportion in PUFA. Fatty acid concentrations, especially in SAFA and MUFA, could be related to physiological condition, sexual development, and recent feeding events. The diet shift revealed by the fatty acid composition suggests changes in habitat use and migration scale in coral reef environment of genus *Abudefduf*.

## Introduction

Malaysia has one of the highest and richest diversity of fish in the world (Arai 2015). Mohsin and Ambak (1996) reported 710 species of marine fishes from the Malaysian waters and their adjacent seas. Furthermore, Ambak et al. (2010) and Chong et al. (2010) listed 2243 and 1951 fish species, respectively, in Malaysian waters. Although several information regarding taxonomy and distribution in coral fish species is available in Malaysian water, few study has done for their life history, ecology and reproduction compared to other coral reef area.

Recently, signature of fatty acid analysis has been increasingly used to study the diet of a number of marine species (e. g. Daly et al. 2010; Stowasser et al. 2012; Couturier et al. 2013). The use of fatty acids as trophic biomarkers is based on the assumption that many fatty acids in the marine environment are characteristic of specific groups (Stowasser et al. 2012). Because animals (e.g. crustaceans and fish as well as humans) cannot synthesize ω-3 and ω-6 fatty acids de novo, they need to obtain these molecules from their diet, and therefore some PUFAs are considered to be essential fatty acids or ‘essential nutrients’ for animals (Parrish 2009). Although some mammals and freshwater fish can synthesize other forms of essential fatty acids, marine fish and freshwater zooplankton have very limited ability for bioconversion (Parrish 2009). These fatty acids can generally not be synthesized in higher trophic levels and are incorporated into tissues of higher trophic individuals (Sargent et al. 1987). Dietary fatty acids are selectively incorporated into different tissues and little is known about which tissue the profile would best mirror the diet profile of fish. It is reported that fish liver can reflect dietary fatty acid within a short timescale (10 weeks) (Beckmann et al. 2013). Thus, fish liver is suitable organ to examine the composition and the fatty acid signature can be useful to trace diet of fish in ecosystem.

The genus *Abudefduf* is commonly found in tropical and subtropical waters that typically occur on coral reefs (Allen 1991). Generally, the fish occurs small groups or singly having highly territorial behavior (Allen 1991; Lieske & Myers 1994). The diet of fish is recognized algae, gastropods, and small crabs (Lieske & Myers 1994). However, diet and feeding ecology of the fish is scarcely understood.

In the present study, fatty acid analyses were used to investigate the trophic ecology of damselfish species *Abudefduf bengalensis* and *A. sexfasciatus* collected in the Malaysian South China Sea. To understand the trophic position in accordance with the growth, fatty acid signatures were compared using size class samples.

## Material and methods

### Fish

**Figure 1:**
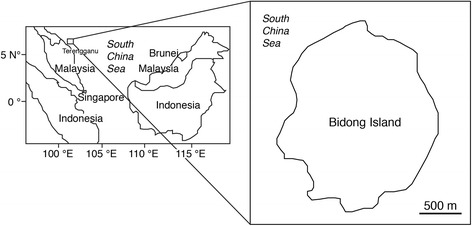
**Map showing the location of the study site at the Bidong Island in Malaysian South China Sea, off the Terengganu State in the east coast of Peninsula Malaysia.**

All specimens of damselfish species *Abudefduf bengalensis* and *A. sexfasciatus* were collected at the Bidong Island in the South China Sea, Malaysia (Latitude 5.62°, Longitude 103.07°) between 27 and 28 October 2014 (Figure 1). Bidong Island is located off Terengganu State on the east coast of Peninsular Malaysia, known for its history as Vietnamese refugee settlement. The island also comprises of being well-developed coral reef ecosystems comprising variety of coral and rocky reef associated fishes (Matsunuma et al. 2011). All fishes were collected by means of fish traps and hook and line. After collecting, all fishes were immediately stored in ice chest, brought back to laboratory, were kept in −20°C freezer and conducted fatty acid analyses within one month. A total of forty-six immature fish samples stage were measured in total length (TL), body weight (BW), and each fish was dissected, liver and the gonad removed to determine their weights (Table 1). Stomach for each fish was dissected for the content analyses.

**Table 1:** **Biological information of genus** ***Abudefduf*** **collected in the Bidong Island Malaysian South China Sea**

	**Total length (cm)**		**Body weight (g)**		**Liver weight (g)**		
**Species**	**mean ± SD**	**range**	**mean ± SD**	**range**	**mean ± SD**	**range**	**N**
*A. bengalensis*	9.4 ± 0.4	8.5 - 10.1	18.6 ± 2.1	14.1 - 23.3	0.07 ± 0.04	0.02 - 0.20	36
(small)							
*A. bengalensis*	14.9 ± 0.6	14.3 - 16.0	93.0 ± 16.8	74.6 - 116.9	1.26 ± 0.33	0.92 - 1.62	5
(large)							
*A. sexfasciatus*	12.9 ± 0.9	11.3 - 14.4	40.7 ± 7.6	31.7 - 53.8	0.35 ± 0.11	0.15 - 0.49	5

N: total number of specimens.

### Fatty acid analysis

Liver samples of *Abudefduf bengalensis* and *A. sexfasciatus* were analysed for fatty acid composition following the one step method (Abdulkadir and Tsuchiya 2008; Arai et al. 2015). Three replicates of each liver and tissue samples were mixed with 4 ml of hexane and 1 ml of internal standard solution in a 50 ml centrifuge tube. After adding 2 ml of 14% BF3 in methanol, the tube was flushed with nitrogen gas. The capped tube was heated on a hot plate at 100°C for 120 min. One ml of hexane was added followed by 2 ml of distilled water. The tube was then shaken vigorously for 1 min and centrifuged for 3 min at 2500 rpm.

Samples were then analysed using a GC-FID (GC 14-B, Shimadzu). Separation was performed with an FFAP-polar capillary column (30 m × 0.32 mm internal diameter, 0.25 μm film thickness). Hydrogen was used as a carrier gas. After injection at 60°C, the oven temperature was raised to 150°C at a rate 40°C min^−1^, then to 230°C at 3°C min^−1^, and finally held constant for 30 min. The flame ionization was held at 240°C. Peaks were identified by comparing their retention times with those of authentic standards (Supelco Inc.). Fatty acids were designated as an n:pωx, where n is the number of carbon atoms in the aliphatic chain, p is the number of double bonds and x is the position of the first double bond from the terminal methyl group. The analytical precision for samples was generally <5% for total amounts and major components.

### Data analyses

Fatty acid concentrations (mg g^−1^ dry weight) were calculated by comparing the peak area of fatty acid in the sample with the peak area of internal standard. The percentage for each fatty acid was converted from the area of chromatogram peaks. The composition is expressed as percentage of total fatty acids (Table 2).

**Table 2:** **Fatty acid composition (mean ± SD) in livers of two species of genus** ***Abudefduf*** **collected in the Bidong Island, Malaysian South China Sea**

**Fatty acids**	***A. bengalensis***	***A. bengalensis***	***A. sexfasciatus***
	**small (n = 36)**	**large (n = 5)**	**(n = 5)**
SAFA			
C14:0	9.1 ± 0.7	6.0 ± 1.8	4.7 ± 0.9
C16:0	48.9 ± 5.3	62.4 ± 9.0	43.4 ± 8.3
C18:0	8.2 ± 7.6	5.6 ± 3.4	1.3 ± 1.7
C20:0	0.5 ± 0.1	0.1 ± 0.1	0.2 ± 0.2
**∑SAFA**	**66.7 ± 2.8**	**74.2 ± 7.8**	**49.5 ± 6.7**
MUFA			
C16:1	8.6 ± 4.2	2.1 ± 1.7	8.7 ± 2.5
C17:1	0.4 ± 0.3	1.1 ± 0.5	0.6 ± 0.2
C18:1ω9c	8.1 ± 1.7	11.6 ± 8.6	30.3 ± 6.5
C18:1ω9t	10.1 ± 2.4	6.0 ± 6.5	7.0 ± 3.0
C20:1	0.1 ± 0.0	0.6 ± 0.8	0.9 ± 1.2
**∑MUFA**	**27.3 ± 6.3**	**21.4 ± 8.0**	**47.4 ± 7.0**
PUFA			
C18:3n3	2.4 ± 1.2	1.6 ± 0.8	1.4 ± 0.2
C18:3n6	0.6 ± 0.3	1.3 ± 0.5	0.4 ± 0.2
C20:3n3	0.1 ± 0.1	0.2 ± 0.1	0.1 ± 0.0
C20:5n3 (EPA)	1.6 ± 0.9	1.3 ± 0.9	0.5 ± 0.2
C22:6n3 (DHA)	1.4 ± 2.2	0.1 ± 0.1	0.6 ± 0.7
**∑PUFA**	**6.0 ± 3.5**	**4.4 ± 1.5**	**3.1 ± 0.8**
**Total concentration (μg/mg)**	**143 ± 56.9**	**169 ± 112**	**273 ± 124**

Differences between data were analysed using the Mann–Whitney *U*-test (Sokal & Rohlf 1995).

## Results

### Biological characteristics

*Abudefduf bengalensis* could catagorise as either small or large according to their sizes (Table 1). Significant differences were found between two size classes in TL (p < 0.0001), BW (p < 0.0001) and liver weight (p < 0.0001).

Stomach content for each fish was observed for ten randomly chosen samples. However, stomach content for each fish could not identify prey organisms under macro- and micro-observations. Thus, we did not conduct stomach content observations for other 36 fishes.

### Difference in fatty acid composition on growth

In *Abudefduf bengalensis*, fatty acid compositions were different between size groups. Proportions of saturated fatty acids (SAFA) ranged 66.7% 74.2%, with the highest proportions in fatty acids (Table 2). Palmitic acid (16:0) was the most common saturated fatty acid ranged from 48.9% to 62.4% (Table 2) followed by C18:0 and C14:0. Significant differences in C16:0, C18:0 and ∑SAFA were found between small fishes (SFs) and large fishes (LFs) (p < 0.05-0.005), however no significant differences were found in C14:0 and C 20:0 (p > 0.05).

Monounsaturated fatty acids (MUFA) were the second dominant ranged from 21.4% to 27.3% (Table 2). Of all MUFA, oleic acid (C18:1ω9c) was the dominant MUFA for all size classes, followed by C16:1 and C18:1ω9t (Table 2). Significant differences in C16:1, C18:1ω9c, C20:1 and ∑MUFA were found between SFs and LFs (p < 0.05-0.0001), however no significant differences were found in C17:1 and C18:1ω9t (p > 0.05).

The proportion of polyunsaturated fatty acids (PUFA) was accordingly low ranged from 4.4% to 6.0% (Table 2). Linolenic acid showed highest ranged from 1.6% to 2.4%, followed by EPA (C20:5n3) and DHA (C22:6n3) (Table 2). Significant differences in C18:3n3 and C20:5n3 were found between SFs and LFs (p < 0.05), however no significant differences were found in other combinations (p > 0.05).

### Difference in fatty acid composition between species

The present study showed growth effect on fatty acid composition in *Abudefduf bengalensis*. Thus, similar size groups were compared between *A. bengalensis* and *A. sexfasciatus* to minimize the size effect on fatty acid composition. Proportion of SAFA in *A. sexfasciatus* was 49.5%, with the highest proportions in fatty acids as same as *A. bengalensis* (Table 2). Palmitic acid was also the most common saturated fatty acid followed by C14:0 and C18:0. Significant differences in C16:0, C18:0 and ∑SAFA were found between those species (p < 0.05-0.01), however no significant differences were found in C14:0 and C 20:0 (p > 0.05).

MUFA were also the second dominant in *A. sexfasciatus* (Table 2). Oleic acid was the dominant MUFA, followed by C16:1 and C18:1ω9t (Table 2). Significant differences in C16:1, C18:1ω9c and ∑MUFA were found between species (p < 0.01-0.0005), however no significant differences were found in C17:1 and C18:1ω9t (p > 0.05).

The proportion of polyunsaturated fatty acids (PUFA) was the lowed averaging 3.1% (Table 2). Linolenic acid showed highest, followed by EPA and DHA (Table 2). A significant difference in C18:3n6 were found between species (p < 0.05), however no significant differences were found in other combinations (p > 0.05).

Total fatty acid concentrations ranged from 143 to 273 μg mg^−1^ (Table 2). No significant differences were found in all combinations (p > 0.05).

## Discussion

It is noteworthy that fatty acid composition was different depending on the size groups in a damselfish species *Abudefduf bengalensis*. Differences in individual fatty acid profiles were reported previously with various factors such as development (Legendre et al. 1995; Soivio et al. 1989), food habits (Daly et al. 2010; Stowasser et al. 2012; Couturier et al. 2013) and habitat use (De Silva et al. 1997; Takeuchi & Watanabe 1982; Tidwell et al. 1992), temperature (Takeuchi & Watanabe 1982) and salinity (Borlongan and Benitez 1992; De Silva et al. 1997). Although the mechanism of lipid deposition in the liver of fish fed diets was still uncertain, fatty acid synthesis was regulated by liver X receptor suggesting the profiles of the liver reflected diets of fish (Peng et al. 2014). The low amount PUFA was found in the present study. In particular, it has been reported that PUFA is able to limit triglyceride deposition in the liver (Xin et al. 2008; Di Minno et al. 2012), whereas a diet deficient in n-3 PUFA with a high n-6/n-3 ratio could induce fatty liver (El-Badry et al. 2007). However, differences in fatty acid composition and levels in relation to body size in wild fish species have not been well reported. It is likely that such differences are caused by differences in the diet, behavior and migration of the damselfish accompanying the growth. The fish were found in coastal and mangrove area during the life (Allen 1991; Lieske & Myers 1994; Matsunuma et al. 2011) The role of mangroves as nursery habitats for some coral fish species has received considerable attention as a link with adjacent coral reef or offshore habitats (e.g. Beck et al. 2001; Parrish 1989; Pollard 1984). The diet shifts of coral reef fish species that inhabit mangroves have been reported their early life stages (Cocheret de la Morinie’ re et al. 2002; Nagelkerken et al. 2000; Cocheret de la Morinie’ re et al. 2003). These findings suggest that differences in fatty acid profile during growth found in the present study might correspond to the diet and habitat shifts in *A. bengalensis*.

SAFA was the most abundant fatty acids and the palmitic SAFA showed highest values among all fatty acids (Table 2). The second most abundant SAFA was stearic acid. These two SAFAs have been reported to have the highest concentrations in other fish species (Elsdon 2010; Sahena et al. 2009), Acetes (Montaño et al. 2001) and in copepods (van der Meeren et al. 2008). The predominance of both fatty acids has been attributed to their use as a major source of energy for metabolism and growth (Sargent et al. 2002). Fishes from warm waters tend to show high levels of palmitic and stearic acids compared to those from cold waters. This difference is due to metabolic differences between cold and warm water species, because these fatty acids are not usually subject to differences in diet (Huynh & Kitts 2009). Damselfish species *Abudefduf bengalensis* and *A. sexfasciatus* were collected in the South China Sea in tropical waters, and thus the fish might have higher palmitic and stearic acid levels in the present study.

MUFA was the second most abundant fatty acids, with highest values for oleic acid (Table 2). This is in agreement with findings in copepod (Olivotto et al. 2010), Acetes (Montaño et al. 2001) and fish fatty acid profiles (Elsdon 2010; Huynh and Kitts 2009; Sahena et al. 2009; Sirot et al. 2008). Oleic MUFA is naturally occurring in large concentrations in many marine organisms, which can also synthesise this MUFA de novo (Sargent et al. 2002). High proportions of MUFAs of marine predators are generally derived from marine zooplankton (Pond & Tarling 2011; Pond et al. 2012). In the present study, we did not conduct fatty acid analyses for potential prey organisms. Nevertheless, the higher level of MUFAs found in *Abudefduf bengalensis* and *A. sexfasciatus* suggest that the fish might feed copepod as one of potential prey organism during the life history.

Fatty acid signature has been increasingly used to study the diet of a number of marine species. The present study suggests that diets of the coral fish species *Abudefduf bengalensis* and *A. sexfasciatus* changed in accordance with growth. Furthermore, differences in fatty acid profiles might not just be considered with respect to the diets, but might be based on the habitat and migration.

### Ethical standards

This study has been conducted in a field station at the Bidong Island belonging to Universiti Malaysia Terengganu. This study was also reviewed and approved by the Universiti Malaysia Terengganu ethics board.
